# Explainable fNIRS-based pain decoding under pharmacological conditions via deep transfer learning approach

**DOI:** 10.1117/1.NPh.11.4.045015

**Published:** 2024-12-17

**Authors:** Aykut Eken, Murat Yüce, Gülnaz Yükselen, Sinem Burcu Erdoğan

**Affiliations:** a TOBB University of Economics and Technology, Biomedical Engineering Department, Ankara, Turkey; b Acıbadem Mehmet Ali Aydınlar University, Department of Biomedical Engineering, Faculty of Engineering and Natural Sciences, Istanbul, Turkey

**Keywords:** functional near-infrared spectroscopy, pain decoding, explainable artificial intelligence, morphine, transfer learning

## Abstract

**Significance:**

Assessment of pain and its clinical diagnosis rely on subjective methods which become even more complicated under analgesic drug administrations.

**Aim:**

We aim to propose a deep learning (DL)–based transfer learning (TL) methodology for objective classification of functional near-infrared spectroscopy (fNIRS)–derived cortical oxygenated hemoglobin responses to painful and non-painful stimuli presented under different timings post-analgesic and placebo drug administration.

**Approach:**

A publicly available fNIRS dataset obtained during painful/non-painful stimuli was used. Separate fNIRS scans were taken under the same protocol before drug (morphine and placebo) administration and at three different timings (30, 60, and 90 min) post-administration. Data from pre-drug fNIRS scans were utilized for constructing a base DL model. Knowledge generated from the pre-drug model was transferred to six distinct post-drug conditions by following a TL approach. The DeepSHAP method was utilized to unveil the contribution weights of nine regions of interest for each of the pre-drug and post-drug decoding models.

**Results:**

Accuracy, sensitivity, specificity, and area under curve (AUC) metrics of the pre-drug model were above 90%, whereas each of the post-drug models demonstrated a performance above 90% for the same metrics. Post-placebo models had higher decoding accuracy than post-morphine models. Knowledge obtained from a pre-drug base model could be successfully utilized to build pain decoding models for six distinct brain states that were scanned at three different timings after either analgesic or placebo drug administration. The contribution of different cortical regions to classification performance varied across the post-drug models.

**Conclusions:**

The proposed DL-based TL methodology may remove the necessity to build DL models for data collected at clinical or daily life conditions for which obtaining training data is not practical or building a new decoding model will have a computational cost. Unveiling the explanation power of different cortical regions may aid the design of more computationally efficient fNIRS-based brain–computer interface (BCI) system designs that target other application areas.

## 1 Introduction

Pain is a vital function of the human body which serves as an early warning signal to protect tissue damage. The extent to which an individual experiences pain still remains a complex and subjective phenomenon and is considered to depend on a variety of intrinsic and extrinsic factors that include the efficiency of communication between the nociceptors and their subcortical and cortical projections^1^ besides genetics, past experiences, and cultural influences. Although the current common methodology for pain assessment relies on self-reports in clinical practice, there may be conditions where patients are unable to provide verbal self-reports such as a surgical procedure performed under anesthesia or in situations where the patient is unconscious due to a variety of conditions such as critical cerebral tissue damage. Patients with severe cognitive impairments or patients who preserve their mental abilities but who cannot communicate with their external environment may also be unable to provide objective and accurate self-reports of their pain experience.

Pain has few biomarkers that can be used in clinical practice.^2^ Some biomarkers are intended to track pain intensity and complement self-reports as a way of assessing the incidence or intensity of pain, whereas others are intended to reveal underlying pathobiological conditions.^2^ However, in the abovementioned situations, there is a lack of an objective biomarker of pain that can aid precise evaluation and management of treatment procedures. Objective identification of pain and non-pain states would have numerous clinical advantages, including the ability to continuously monitor and assess neural correlates of pain intensity during surgery and quantitative evaluation of the progress and efficacy of a treatment strategy. Such an objective evaluation marker could assist the execution of operational procedures under optimal conditions through adjustment of the analgesic regime when required.

Previous functional neuroimaging studies conducted with positron emission tomography (PET), functional magnetic resonance imaging (fMRI), and functional near-infrared spectroscopy (fNIRS) demonstrated consistent pain-related localized hemodynamic responses in the human brain.^3^^–^^5^ Moreover, these studies also demonstrated spatial and temporal differences in the neural processing of low- and high-intensity painful stimuli.^3^^–^^6^ Pain-induced deactivation in the medial prefrontal cortex (mPFC) regions during both acute and chronic conditions has also been consistently observed across different neuroimaging studies conducted with different modalities.^7^^–^^9^ Moreover, morphine-induced attenuation of the deactivation in the mPFC during the processing of painful stimuli was also reported in the study of Peng et al. (2018).^19^ Overall, these studies have provided valuable insights into the neural mechanisms underlying pain processing in the human brain. Besides, they also addressed the promise of exploring robust biomarkers of pain processing under analgesic or different drug administrations.

Among these modalities, fNIRS has shown a great potential for extracting objective biomarkers of pain in clinical or operative environments due to its numerous advantages such as the ability to collect hemodynamic data non-invasively with wearable ergonomic probes that can be placed at the surface of the scalp. Previous studies using fNIRS have consistently demonstrated significant changes in oxygenated hemoglobin (HbO) concentration in the prefrontal cortex (PFC) in response to painful stimuli, including cutaneous,^10^ dental,^11^ and visceral pain.^12^ These observations are supported by findings from fMRI studies reporting deactivations in anterior PFC blood oxygenation level–dependent signals following painful stimuli.^13^^–^^16^ Under the analgesic state, Beccera et al.^12^ obtained hemodynamic recordings from the PFC during a colonoscopy procedure. Analysis of fNIRS data revealed a specific, reproducible PFC activity corresponding to the time intervals when patients grimaced. The pattern of activation was similar to that obtained in previous studies in awake healthy individuals, whereas they were exposed to nociceptive stimuli. Similar hemodynamic activation patterns obtained during painful events under both awake and sedative conditions suggest that unsuccessful inhibition of the neuronal processing of a nociceptive stimulus due to insufficient levels of analgesia can be objectively quantified with fNIRS-derived biomarkers. Karunakaran et al.^17^ also showed that the use of fNIRS during knee surgery can provide objective measures of pain-related brain activity. After analyzing fNIRS data obtained during pre-, intra-, and post-operative stages, they found a decrease in resting-state functional connectivity (FC) within the mPFC during the post-operative state when compared with the preoperative awake state. Also, they observed that negative intraoperative FC between the mPFC and somatosensory cortex (S1) was associated with higher reported post-operative pain levels. As a conclusion from this study, it can be inferred that neurophysiological information obtained from fNIRS recordings during surgery can provide objective measures of pain-related brain activity. In a study by Kussman et al.^18^ involving patients undergoing catheter ablation of arrhythmias, somatosensory and frontal cortical hemodynamic activations were measured with fNIRS. The results showed that a decrease in HbO concentration in response to the ablative lesions was observed in the frontal cortical regions. These cortical signals mirrored the responses seen in awake, healthy volunteers and findings from other studies involving nociceptive stimulation. These studies highlight the feasibility and potential utility of fNIRS as an objective measure of cortical activation during nociceptive procedures under general anesthesia.

Despite the promising results, there are challenges in using fNIRS-derived neural markers for accurate detection of pain. One issue is the presence of habituation effect which results in a decrease in the amplitude of hemodynamic responses to repeated painful stimuli over time.^5^ In addition, the shape of the hemodynamic response function obtained during painful stimuli presents intra- and intersubject variability which has also been shown to be dependent on the cortical regions and stimuli types.^5^ One major limitation for deriving robust pain biomarkers from both fNIRS or fMRI signals via mass univariate statistical approaches relies on the low spatial and functional sensitivity of these techniques because the achieved spatial resolution spans millions of neurons with diverse functional properties and distributed connections across different layers. Another issue that has been noted in the use of fNIRS for the detection of pain is the effect of analgesics, specifically opioids such as morphine, on the hemodynamic response. Peng et al.^19^ found that morphine administration was associated with an attenuated HbO signal in the medial portion of the anterior prefrontal cortex (Brodmann Area 10) in response to painful stimuli.

Evaluation of hemodynamic and behavioral correlates of pain is performed by the use of conventional statistical approaches which provides insight into the population level and does not allow inferences to be made at the single subject or single stimulus level. Due to these limitations, accurate detection and objective identification of pain and non-pain states under different pharmacological conditions are challenging problems. Within this context, deep learning (DL) techniques may provide a more effective approach to the problem of detecting pain and non-pain states from information obtained with functional neuroimaging modalities. DL methodologies provide several benefits such as the integration of all available biological data into a single “best prediction” about the output of the algorithm besides their ability to capture information across multiple spatial scales.

Several studies implemented DL methods to fMRI and fNIRS signals to search for “fingerprints” specific to acute pain processing. Rojas et al.^20^ aimed to develop an objective tool for assessing pain in non-verbal patients using DL models and fNIRS data. The authors explored the utility of different DL models and compared their performance in the accurate identification of pain. The study found that a combination of forward and backward information in the bidirectional long short-term memory (Bi-LSTM) model achieved a 90.6% accuracy in two-class classification of pain intensity level. The use of DL models eliminated the need for complex feature extraction procedures and reduced subjectivity associated with extracting hand-crafted features when compared with supervised machine learning models. These findings represented a step forward in the development of a physiologically based diagnosis of pain and can assist clinicians in the objective assessment of pain in non-verbal patients. Pain assessment via fNIRS was also carried out using machine learning (ML) techniques. Lopez-Martinez et al.^21^ tested the efficacy of combining Bayesian hierarchical modeling with scalogram-based features of HbO signals to decode the presence of painful or non-painful stimuli. Their binary classification achieved 81% accuracy with 75% precision. Another study focused on decoding low- and high-intensity pain under two different temperature levels (cold and hot).^22^ Features from time, frequency, and wavelet domains were utilized to train support vector machine (SVM), K-nearest neighborhood (KNN), and linear discriminant analysis (LDA) classifiers. They achieved a four-class classification accuracy of 94.17% with 25 features. Another recent study focused on decoding hemodynamic responses obtained during no pain, low pain, and high pain conditions using both HbO and deoxyhemoglobin (HbR) concentration changes.^23^ By fusing statistical features obtained from both signals, the authors achieved a three-class classification accuracy of 68.51%±9.02%.

The aim of this study was to propose a deep learning (DL)–based transfer learning (TL) methodology for objective and accurate classification of fNIRS-derived cortical HbO responses to painful and non-painful stimuli that were presented under different timings post-analgesic and placebo drug administration. TL is a specific supervised learning method that involves the transfer of knowledge (i.e., feature weights) from a pre-trained base model to a new model that is utilized to make inferences about a similar population data after the addition of a few computationally efficient fine-tuning steps.^24^ Within the context of the proposed work, the TL approach was utilized to transfer knowledge of the constructed DL model from pre-drug fNIRS scans, and the base neural network knowledge of the pre-drug DL model was adapted to the problem of binary classification of hemodynamic responses to painful and non-painful stimuli collected under two different drug administrations (i.e., morphine and placebo) and at three-time points post-drug administration (i.e., 30, 60, and 90 min).^19^

## 2 Materials and Methods

### 2.1 Dataset

An fNIRS dataset that was previously published in Ref. 19 was utilized in the presented work. Fourteen male volunteers (mean ± standard deviation: 29±5 years) who had no recent history of pain or opioid abuse were recruited. Each subject had two site visits where he was administered either an oral morphine or a placebo pill. At each site visit, fNIRS scans were taken during a nociceptive stimuli protocol (a) before and (b) after administration of an oral morphine or a placebo pill. The pills looked identical, and the order of placebo or morphine administration was randomized.

At each site visit, subjects had an fNIRS scan prior to drug administration during the nociceptive stimuli protocol which consisted of six painful and six non-painful stimuli that were delivered to the left thumb with an electrical stimulator. Low-level pain and high-level pain conditions were explained as two distinct scores 3 and 7 over a 0 to 10 scale; a 3/10 score was described as “subject is strongly aware of stimulus but does not perceive any pain,” and a 7/10 score was described as “subject perceives high levels of pain, but it should be tolerable without breath holding or any retreat actions.”^19^ For each subject, the electrical stimulus intensities that corresponded to painful and non-painful conditions were determined prior to the pre-drug session, and they remained the same across the post-drug sessions.

Each nociceptive stimulus lasted for 5 s followed by a 25-s rest period. The same nociceptive stimuli paradigm was applied to participants at separate fNIRS sessions that took place after 30 min [Post-Morphine-30 (Post-MM-3]), 60 min [Post-Morphine-60 (Post-MM-60)], and 90 min [Post-Morphine-90 (Post-MM-90)] of morphine administration and after 30 min [Post-Placebo-30 (Post-PM-30)], 60 min [Post-Placebo-60 (Post-PM-60)], and 90 min [Post-Placebo-90 (Post-PM-90)] of placebo administration. fNIRS recordings were collected from the medial portion of the frontopolar cortex (medial Brodmann Area 10), the right primary S1, and a portion of the left lateral PFC.

### 2.2 Regional Information

The publicly available fNIRS dataset included real head coordinates of source and detector positions for each subject and scan. These real head coordinates were converted to Montreal Neurological Institute (MNI) coordinates through the Colin27 Atlas^25^ by use of the NIRS-SPM toolbox^26^ to reveal the corresponding cortical region. The head coordinates of individual optodes and channels were extracted for each drug administration scan of each subject. After estimation of the MNI coordinates from real head coordinates, the MNI coordinates of the pre-scan session of morphine and placebo administration of 14 subjects were averaged for each optode position. Supplementary Material 1 demonstrates the mean MNI coordinates of each channel and the relevant standard deviation across subjects averaged across all scans.^27^ After spatial registration of optode coordinates to the MNI space, 10 cortical regions were determined which included the right primary motor cortex (R MI), right somatosensory cortex (R SI), right and left pre motor cortices (R & L PMC), left inferior frontal gyrus (L IFG), right and left frontopolar area (R & L FPA), right and left dorsolateral prefrontal cortices (R & L DLPFC), and right supramarginal gyrus (R SMG). For morphine administration scans, the 10 cortical regions of interest were determined as R MI, R SI, R & L PMC, L IFG, R & L FPA, R & L DLPFC, and R SMG. For the placebo session, real coordinates corresponded to nine cortical regions of interest that included R SI, L the IFG, R & L PMC, R & L FPA, R & L DLPFC, and R SMG. As the morphine and placebo sessions were conducted at different times for each subject, slight variations in cap placement at the subject level may have occurred between the two sessions which may have caused slight changes in optode coordinates across the sessions. In addition, group-level averaging of each channel coordinate per session may also have caused variations in attributing each channel to a specific region of interest (ROI) in Colin27 Atlas which may have resulted in missing R MI region for the placebo sessions.

### 2.3 Dataset Preparation

#### 2.3.1 Data preprocessing and trial extraction

fNIRS data preprocessing was performed with the HomER3 toolbox.^28^ Light intensity data were first converted to optical density (OD). Motion artifacts were removed from OD data by a hybrid approach where wavelet transform^29^ and principal component analysis^30^ were applied consecutively to preserve as many trials as possible unlike the process followed in the original study from which the data were borrowed (Peng et al., 2018). After motion artifact removal, a Butterworth band-pass filter with high and low cutoff frequencies of 0.01 and 0.1 Hz, respectively, was applied to remove heart beat (>1  Hz), respiration (0.15 to 0.4 Hz),^31^ and Mayer waves (∼0.1  Hz).^32^  HbO and ΔHbR concentration changes (ΔHbO and ΔHbR, respectively) were computed using the modified Beer–Lambert law.^33^ For each channel ΔHbO signal, the preprocessed ΔHbO signal of the closest short channel was utilized as a regressor to linearly regress out the systemic physiological interferences that were embedded in the long channel. Let S and L represent the time series ΔHbO data at short and long channels consequently, respectively. To perform a linear regression of systemic noise from long channels, the beta coefficients of short channels ($\beta_{\text{short}}$) were estimated using the equation below

$$\beta_{\text{short}}=(S^{T}S{)}^{-1}S^{T}L.$$ (1)

The scaling coefficient $\beta_{\text{short}}$ was used to linearly regress out the non-neuronally induced systemic physiological effects recorded by short channels using the following equation:

$$L^{\prime }=L-\beta_{\text{short}}\times S.$$ (2)

For each pain or non-pain trial, a pre-stimulus period of 1 s and a 30-s period after the onset of each stimulus (i.e., 5 s of electrical stimulus application and 25 s of resting period) were truncated. Each trial block was down-sampled to 1 Hz to reduce the computational complexity during model training. For each subject, these data were organized in a matrix named D with dimensions set as number of trials (N) × number of time points (T) × number of channels (C) for “painful” or “non-painful” stimuli classes.

For each experimental session, the time series data represented as D were reorganized as $D=\{(X_{1},Y_{1}),(X_{2},Y_{2}),\ldots \ldots \ldots ,(X_{N},Y_{N})\}$, where $X_{i}$ is a three-dimensional matrix with dimensions of number of trials × number of time points × number of channels for each subject i and Y represents the corresponding stimulus intensity (i.e., painful or non-painful Y={−1,1}) for each element of $X_{i}$. As two pre-drug sessions existed for each subject, 336 labeled trials (i.e., 2 sessions × 14 subjects × 6 trials × 2 intensity levels) were obtained from the pre-drug sessions, and 168 labeled trials (i.e., 14 subjects × 6 trials × 2 intensity levels) were obtained from each post-drug session (i.e., Post-MM-30, Post-MM-60, Post-MM-90, Post-PM-30, Post-PM-60, and Post-PM-90). Hence, the feature matrix had dimensions of 336×31×24 for each pre-drug session and 168×31× 24 for each post-drug session.

### 2.4 Deep Learning Steps

**Fig. 1:**
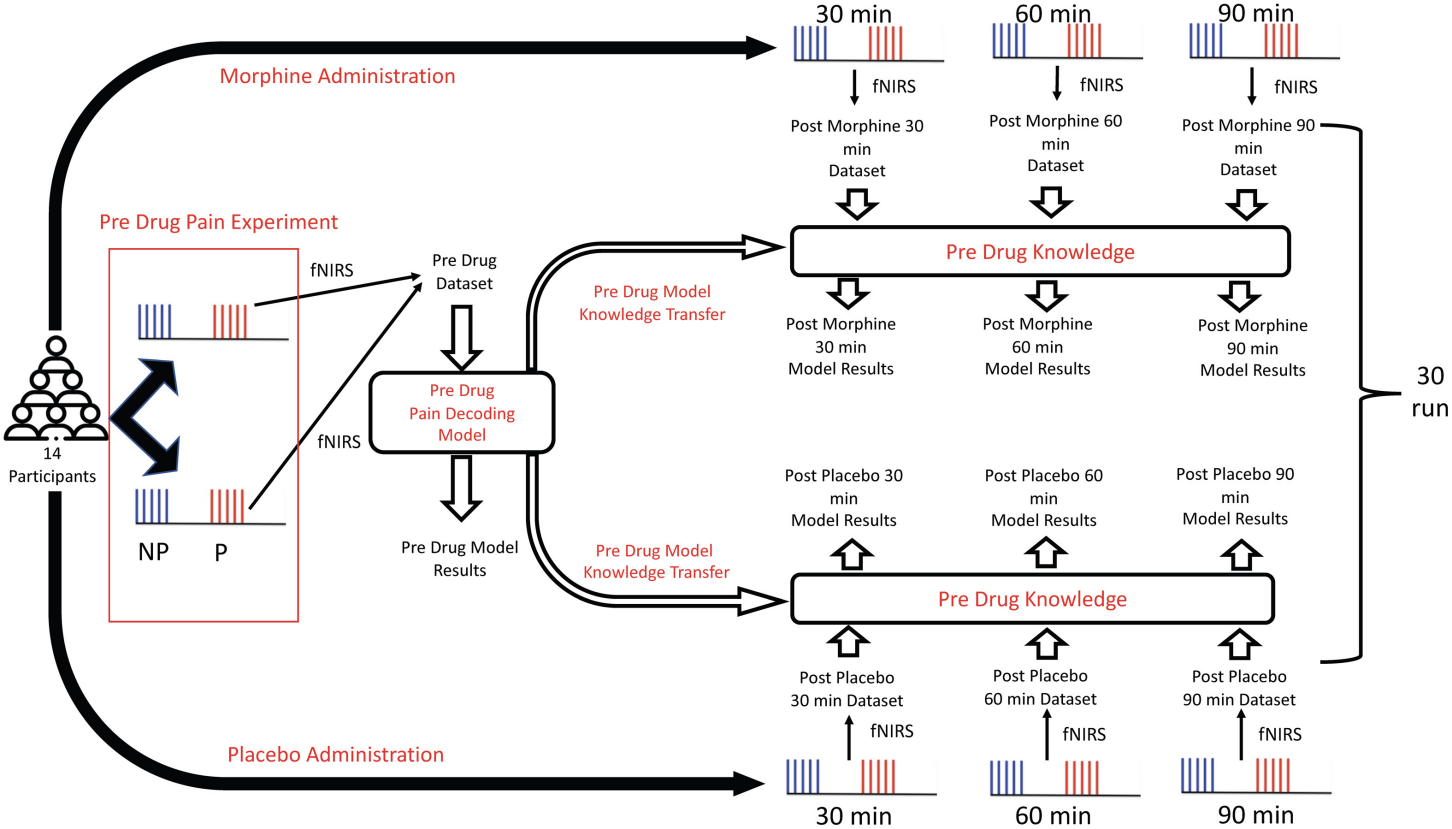
Pipeline of the analysis steps. P, pain; NP, non-pain.

After preprocessing and reorganization of the fNIRS time series data, the DL model training steps included (i) data augmentation, (ii) implementation of the deep neural network (DNN) architecture design, and (iii) adapting the TL approach to post-drug datasets. During DNN training, only ΔHbO data were utilized due to higher SNR compared with ΔHb.^34^ The Tensorflow toolkit (version 2.8.0)^35^ was utilized to construct and design the DNN architecture and for further application of the TL approach to each of the six post-drug datasets. This procedure was repeated 30 times by randomizing the data augmentation step (the details of this step are explained in Sec. 2.4.1) and averaging all loss and accuracy results. The pipeline depicting the order of analysis steps is shown in Fig. 1.

#### 2.4.1 Data augmentation

The pre-drug dataset for constructing the baseline model was split into 60% training, 20% test, and 20% validation sets. At each run, training, test, and validation datasets were randomized. Data augmentation was performed due to the relatively small sample size of the available fNIRS data when compared with other application areas of DL (e.g., automation, finance). For the data augmentation procedure, time-domain approaches were applied to each truncated ΔHbO trial time series which involved either the addition of a linear trend or Gaussian noise.^36^ The linear trend addition procedure involved the addition of linear trends whose slope values were randomly chosen as 0.01, 0.05, or 0.1. These slope values were randomly selected and added to the truncated ΔHbO time series of each trial of each channel. The second approach involved the addition of Gaussian noise with zero mean and randomly selected variance (0.01, 0.05, and 0.1) to each trial time series data of each channel. After pooling single trial ΔHbO data from all channels and subjects [i.e., 336 trial data (2 sessions × 14 subjects × 6 trials × 2 stimulus intensity levels) × 31 time points × 24 channels], the training portion of this dataset was augmented 25 times with randomized application of either of the abovementioned time-domain procedures.

#### 2.4.2 Proposed DNN architecture

**Fig. 2:**
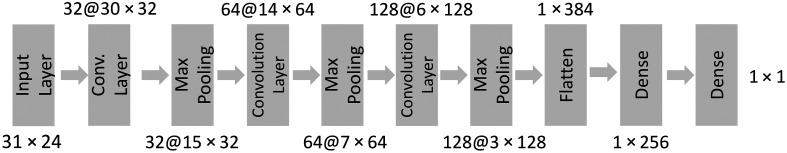
Flowchart of the proposed DNN structure. Numbers given on the top or bottom of each layer indicate the filter size of the layer @ output size of the layer.

A DNN based on one-dimensional (1D) convolutional layers was developed. In this network, three 1D convolutional layers existed whose filter counts were 32, 64, and 128 with a convolution length of 2. Rectified linear unit (ReLU) layers were added as the activation function to the output of these layers. 1D max-pooling layers with a window size of 2 were added to the output of these ReLU layers. A drop-out layer with a rate of 0.4 was added after every max pooling layer to avoid overfitting. After the third dropout layer, a flattened layer was introduced to transform the output obtained from the last dropout layer into a one-dimensional vector instead of a two-dimensional one. The flattening layer was followed by the addition of a dense layer with 256 units, a ReLU activation function, and an additional dropout layer with a rate of 0.4. The final output layer consisted of the classification layer with a sigmoid function. A graphical representation of the designed network and its summary from Tensorflow are shown in Fig. 2.

Training the pre-drug model (Pre-DM) involved the use of the Adam optimizer with a learning rate of $\eta =10^{-4}$. During each training session, a dynamic learning strategy was applied where the learning rate was reduced with a factor of 0.01 if validation loss did not change during 10 consecutive epochs, and this strategy continued till the minimum η became $10^{-6}$. The batch size was 16, and the number of epochs was 100. For the post-drug models (i.e., Post-MM and Post-PMs), training of added layers was carried out using the Adam optimizer with a learning rate of $\eta =10^{-4}$, and similar to the Pre-DM training, the learning rate was reduced with a factor of 0.01 if validation loss did change during 10 consecutive epochs and/or till a minimum η of $10^{-6}$ was obtained. The batch size for post-drug models was also 16, and the number of epochs was 100.

#### 2.4.3 Fine-tuned TL approach

TL is a relatively new approach for developing fNIRS-based BCI models.^37^ It is based on the premise that knowledge generated from a pre-trained base model can be used to solve another similar classification problem on a novel dataset.^24^ The Pre-DM was constructed with fNIRS HbO data recorded during pre-drug sessions. Our purpose was to transfer knowledge generated from this pre-model to construct post-drug (Post-DM) models obtained under two different pharmacological conditions (i.e., Post-MM and Post-PM) and at three time points post-drug administration (30, 60, and 90 min). Similar to the Pre-DM, the post-drug models took fNIRS signals collected during the same nociceptive paradigm as input. The rationale behind utilizing a TL approach relies on the assumption that such an adaptive training methodology would be able to capture the common neural signature of painful and non-painful stimuli obtained during dynamic brain states which were altered by a pharmacological intervention, and this alteration would be expected to change with respect to time.

After training the Pre-DM, the attained weights (i.e., knowledge) obtained among the pre-trained layers that began from the first convolutional layer to the last max-pooling layer were transferred to construct post-drug models. For fine-tuning purposes, an additional flattened layer, a dense layer with 256 units, a dropout layer with a rate of 0.4, and a final classification layer with a sigmoid activation function were adjusted for each of the post-drug decoding networks separately. The final feature information was utilized to predict the label of stimuli (painful/non-painful) obtained during post-morphine and post-placebo fNIRS scans of 30-, 60-, and 90-min post-drug administration sessions. The accuracy, sensitivity, and specificity results are reported as an average of 30 runs. Data corresponding to painful stimuli were labeled as positive (+) class, and non-painful stimuli data were labeled as negative (−) class.

### 2.5 DeepSHAP Explanation

The Deep SHapley Additive exPlanations (DeepSHAP) method^38^ was adapted to each model to evaluate the contribution of different cortical regions to model specific decoding performance. The SHAP approach is based on estimating a parameter named Shapley value which basically estimates the relative contribution of a feature to an output when compared with all possible other feature combinations.^39^ The DeepSHAP approach is defined as the integration of the SHAP method into the DeepLIFT algorithm to understand the feature-specific contribution to the final classification decision.^40^ The output of a neural network is decomposed to each input by performing backpropagation of neuronal contributions to every feature, and SHAP values are estimated based on the independence assumption of input features and linearity of the model.

Within the context of the proposed work, the contribution of each input feature (feature set: no. of channels × no. of time points per trial) to the final decision of the network was computed for every run, and the contribution of features extracted from all channels within each defined ROI (Supplementary Material 1) was defined as the Shapley contribution of the relevant ROI. Therefore, a Shapley value matrix with dimensions of number of runs × number of ROIs was computed for each of the Pre-DM and post-drug models. After every model training, Shapley values were estimated using DeepSHAP. For the DeepSHAP explainer of Pre-DM, test data had a size of 67 (no. of trials) × 31 (no. of time points) × 24 (no. of channels). At each run, 67 test samples included data from both classes. Shapley values across all channels within each ROI were averaged to interpret the independent contribution of each ROI to classification performance. For post-drug sessions, the corresponding test data for estimating Shapley values had a size of 33 (no. of trials) × 31 (no. of time points) × 24 (no. of channels).

### 2.6 Statistical Analysis

The accuracy, sensitivity, and specificity performances of Pre-DM and Post-DMs were compared based on values obtained from 30 runs. For each performance metric, the normality of performance results from all models was tested using the Shapiro–Wilk test. Because the distribution of values belonging to each of the performance metrics violated the normality assumption, the statistical comparison between Pre-DM and Post-DM performances was carried out using the Kruskal–Wallis test for accuracy, sensitivity, and specificity metrics. Post hoc comparisons were conducted with Bonferroni.

The classification performance of Post-DMs was compared using a 2×3 ([morphine, placebo] × [30, 60, and 90 min]) repeated measures analysis of variance (ANOVA) after performing a Box–Cox transformation on all results due to non-normal distribution of data samples belonging to each of the performance metric.

## 3 Results

### 3.1 Deep Transfer Learning Model Performances

**Fig. 3:**
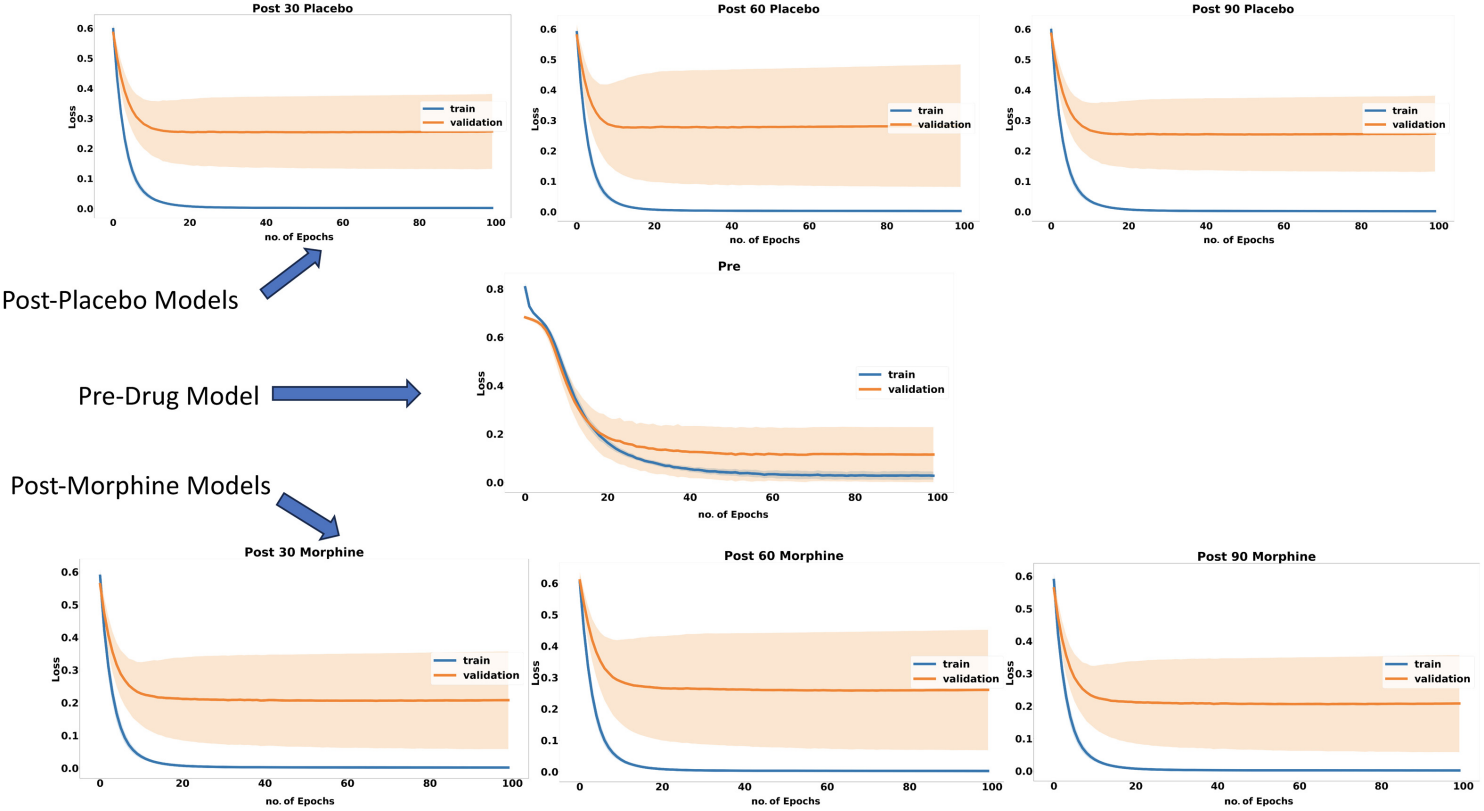
Loss curves of each model during training and validation phases.

**Fig. 4:**
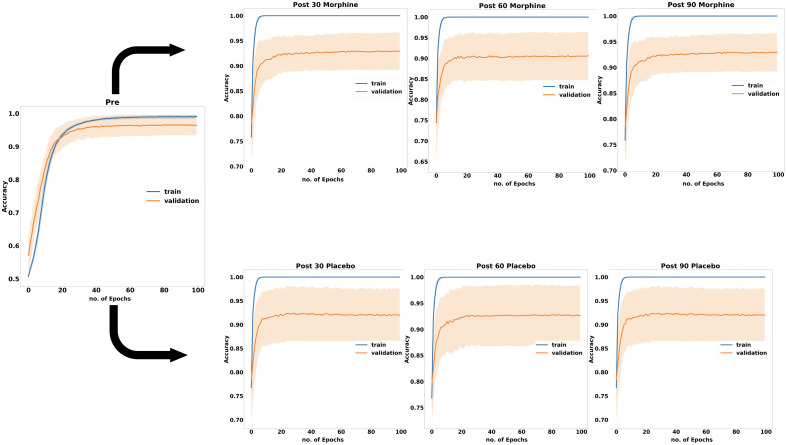
Accuracy performance profiles of each model on training and validation data during the training phase.

Loss curves of each model during the training and validation phases are presented in Fig. 3, whereas accuracy performance profiles of each model for training and validation data during the training phase are demonstrated in Fig. 4. The final training and validation accuracy scores of 30 runs reached to 0.99±0.003 and 0.97± 0.02 for Pre-DM. The training accuracy values reached 1.0 after 10 to 15 epochs for the post-drug models. For Post-PM models, validation accuracies of Post-PM-30, Post-PM-60, and Post-PM-90 reached 0.91±0.05, 0.90±0.05, and 0.92±0.05, respectively. For Post-MM models, validation accuracies of Post-MM-30, Post-MM-60, and Post-MM-90 reached 0.92±0.06, 0.90±0.06, and 0.92±0.06, respectively.

Test performances of Pre-DM and all Post-DMs in terms of their accuracy, sensitivity, and specificity results are given in Table 1. The accuracy, sensitivity, specificity, and AUC metrics of the Pre-DM were above 90%, whereas each of the post-drug models demonstrated a binary classification performance above 90% for the same metrics.

**Table 1:** Test performances of Pre-DM and all Post-DMs in terms of their accuracy, sensitivity, and specificity results averaged across 30 runs.

Session		Accuracy (mean Std. Dev.)	Sensitivity (mean Std. Dev.)	Specificity (mean Std. Dev.)	AUC (mean Std. Dev.)
Pre-drug		0.97 ± 0.03	0.97 ± 0.04	0.97 ± 0.04	0.97 ± 0.03
Morphine	30 min	0.91 ± 0.05	0.90 ± 0.08	0.91 ± 0.05	0.91 ± 0.05
	60 min	0.90 ± 0.07	0.88 ± 0.11	0.90 ± 0.07	0.90 ± 0.07
	90 min	0.91 ± 0.05	0.89± 0.08	0.91 ± 0.05	0.91 ± 0.05
Placebo	30 min	0.92 ± 0.06	0.92 ± 0.08	0.92 ± 0.06	0.92 ± 0.06
	60 min	0.92 ± 0.05	0.91 ± 0.08	0.92 ± 0.05	0.92 ± 0.05
	90 min	0.91 ± 0.07	0.91 ± 0.08	0.91 ± 0.07	0.91 ± 0.07

**Fig. 5:**
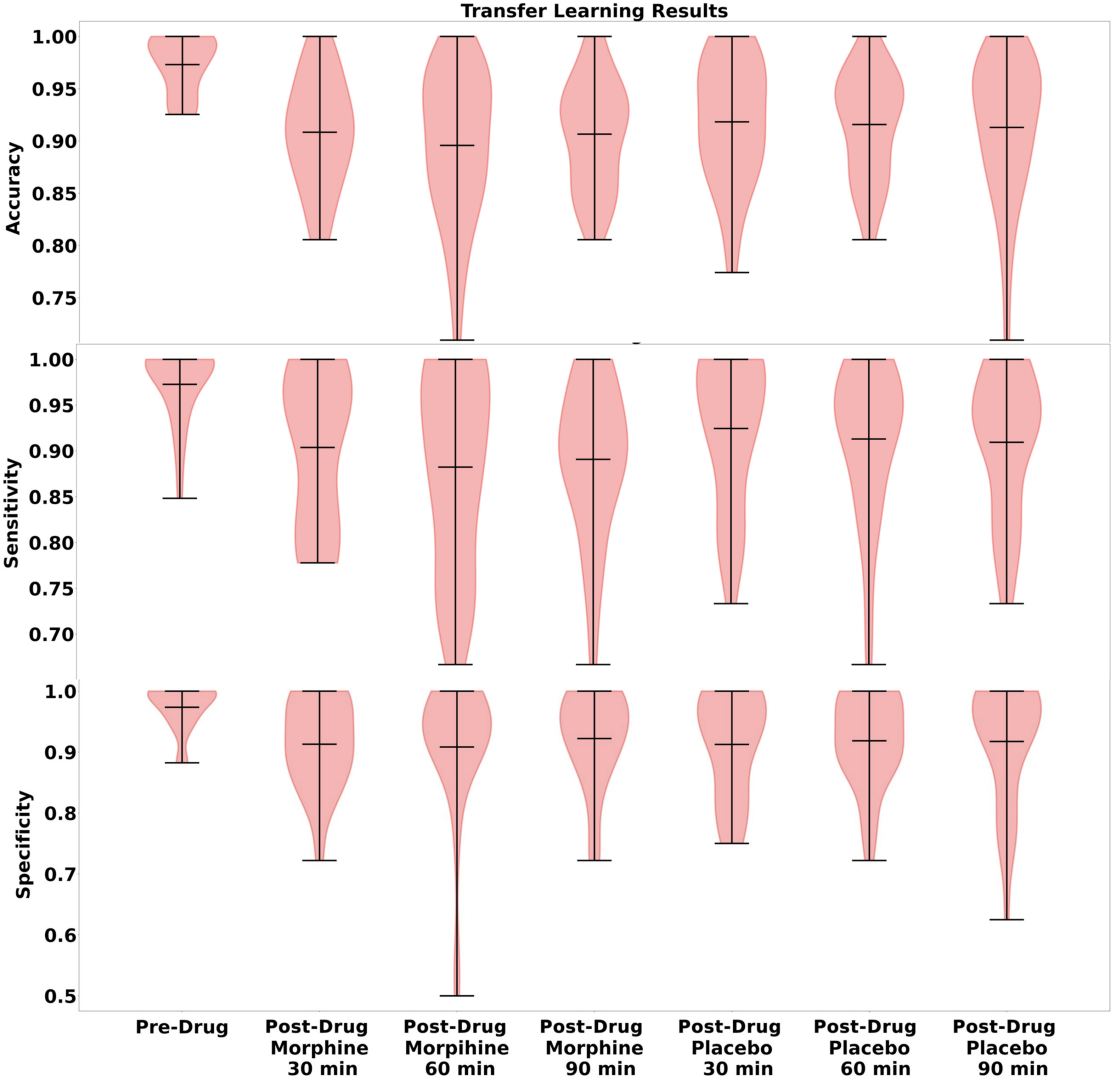
Violin plots of accuracy, sensitivity, and specificity results of Pre-DM, Post-PM, and Post-MM models.

Figure 5 presents violin plots of accuracy, sensitivity, and specificity performances of all models.

### 3.2 Statistical Comparison of Model Performances

#### 3.2.1 Pre-drug model versus post-drug models

The Kruskal–Wallis test results revealed that there is a statistically significant difference between Pre-DM and Post-MMs in terms of accuracy, sensitivity, and specificity scores. Similarly, a statistically significant difference existed between Pre-DM and Post-PMs in terms of accuracy, sensitivity, and specificity scores (Table 2).

**Table 2:** Kruskal–Wallis test results.

Metric	Pre-DM versus Post-MM [$\chi^{2}$ (DF = 3)]	Pre-DM versus Post-PM [$\chi^{2}$ (DF = 3)]
Accuracy	35.41**	27.24**
Sensitivity	20.04**	15.13*
Specificity	15.68**	11.44*

DF, degrees of freedom.

* p<0.05.

** p<0.001.

Multiple comparison tests using Bonferroni correction showed that Pre-DM accuracy results were significantly higher than Post-MM-30, Post-MM-60, and Post-MM-90 (Table 3). Similarly, Pre-DM sensitivity results were found to be significantly higher than Post-MM-30, Post-MM-60, and Post-MM-90, whereas Pre-DM specificity results were found to be significantly higher than Post-MM-30 and Post-MM-60 but not Post-MM-90. No significant differences were found across post-MMs for any of the performance metrics.

**Table 3:** Post hoc statistical comparison of the performance metrics for each Post-Drug Model relative to pre-DM.

Performance metric	Pre-DM versus Post-MM-30	Pre-DM versus Post-MM-60	Pre-DM versus Post-MM-90	Pre-DM versus Post-PM-30	Pre-DM versus Post-PM-60	Pre-DM versus Post-PM-90
Accuracy	41.55**	45.66**	42.91**	37.06**	37.07**	38.05**
Sensitivity	27.30*	34.05**	33.45**	24.23*	24.53**	31.90*
Specificity	27.30*	34.05**	21.83	26.10*	22.93*	22.30

* p<0.05.

** p<0.001.

Pre-DM accuracy results were found to be significantly higher than Post-PM-30, Post-PM-60, and Post-PM-90. Pre-DM sensitivity results were also significantly higher than each post-PM. Pre-DM specificity results were significantly higher than Post-PM-30 and Post-PM-60 models but not Post-PM-90. No significant differences were found across Post-PMs for any of the performance metrics (Table 3). The violin plots depicted in Fig. 5 provide a comparative illustration of the distribution of classification performance metrics of Pre-drug and Post-drug models.

#### 3.2.2 Classification performance comparison of post-drug models

2×3 repeated measures ANOVA for accuracy scores revealed a significant main effect of drug type (F(1,179)=9.98, p=0.002). Statistical significance for neither the main effect of time condition (F(2,179)=0.1, p=0.901) nor the interaction between drug and time conditions (F(2,179)=0.65, p=0.525) was found. Multiple comparisons for the main effect of drug type revealed that placebo condition showed significantly higher accuracy performance than morphine condition (mean difference −0.0198, p=0.002). 2×3 repeated measures ANOVA for sensitivity scores revealed no significance for the main effect of drug type (F(1,179)=3.22, p=0.070), time (F(1,179)=0.62, p=0.540), or a drug and time interaction (F(1,179)=0.08, p=0.922). For specificity results, similarly, no significant main effect for drug type (F(1,179)=0.03, p=0.859), time (F(1,179)=0.1, p=0.901), or a drug and time interaction (F(1,179)=0.23, p=0.791) was found.

#### 3.2.3 Shapley interpretation

**Fig. 6:**
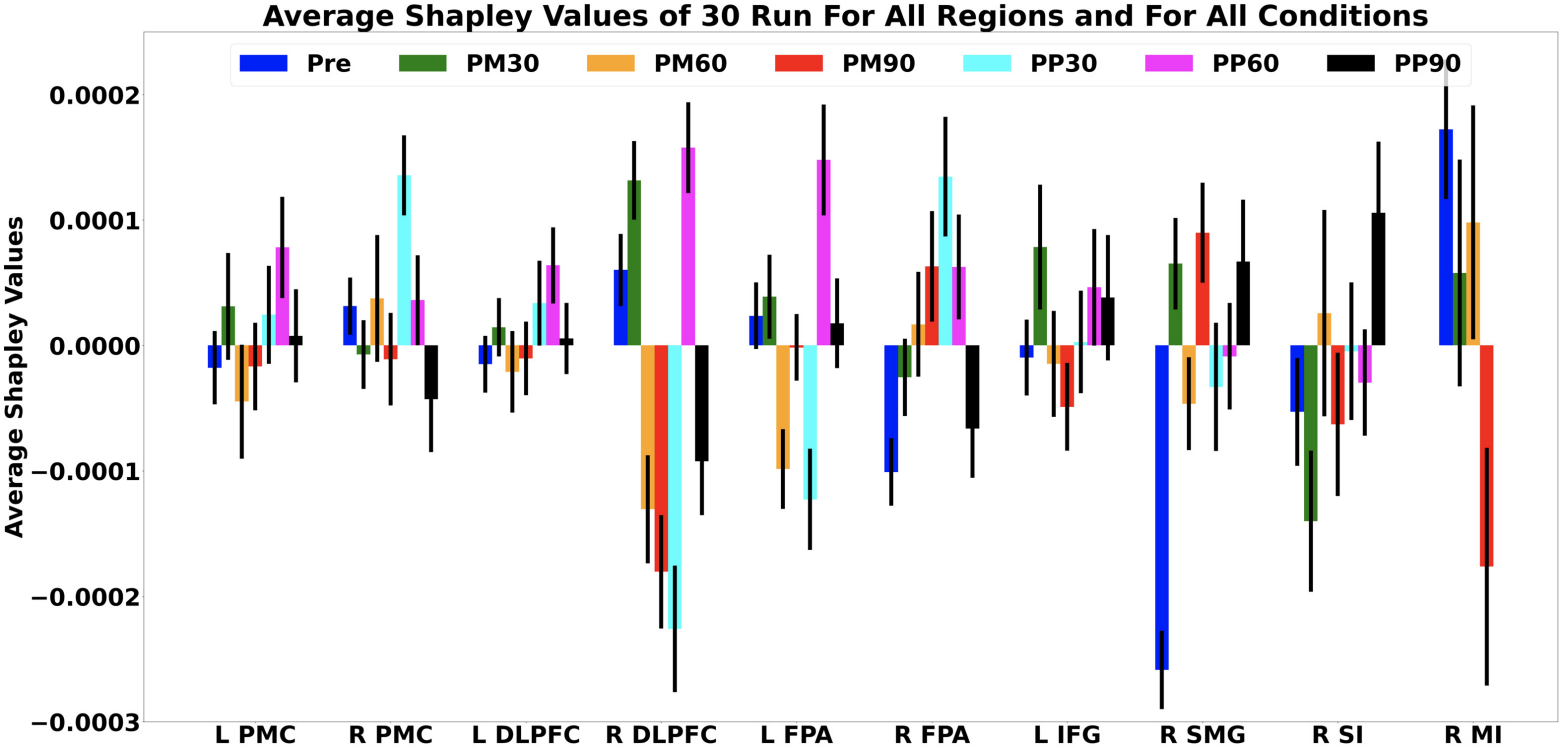
Average Shapley contributions of each ROI to Pre-DM and all Post-DMs across 30 runs. Pre, pre-model; PM30, post-morphine 30 min; PM60, post-morphine 60 min; PM90, post-morphine 90 min; PP30, post-placebo 30 min; PM60, post-placebo 60 min; PM90, post-placebo 90 min; L, left; R, right; PMC, pre-motor cortex; DLPFC, dorsolateral pre-frontal cortex; FPA, frontopolar area; IFG, inferior frontal gyrus; SMG, supramarginal gyrus; SI, somatosensory cortex; MI, motor cortex. Error bars present the standard deviation of the mean.

**Fig. 7:**
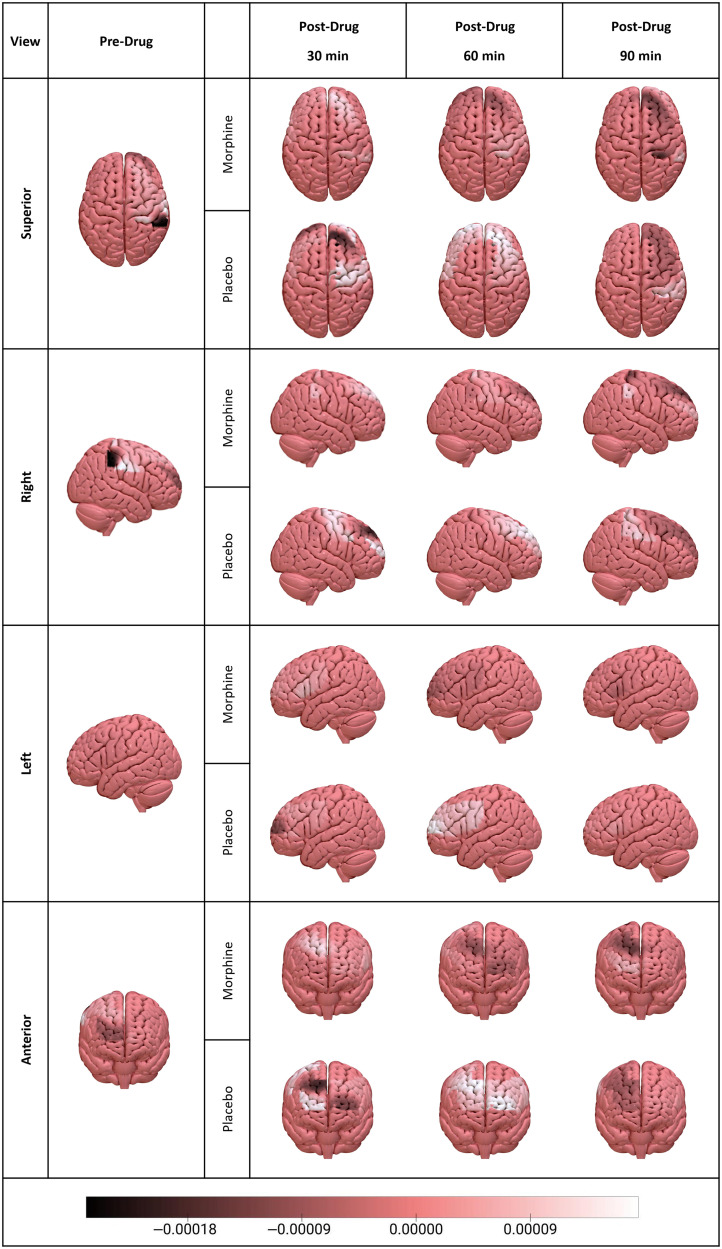
Cortical projection of Shapley contributions of different ROIs to Pre-DM, Post-MM, and Post-PMs.

The sign of Shapley value of an ROI is indicative of whether that ROI contributes positively or negatively to the general decoding performance of a model. The average Shapley contributions of each ROI to Pre-DM and all Post-DMs are plotted in Fig. 6, and their cortical projections are schematically demonstrated in Fig. 7. A list of ROIs that have positive Shapley contributions to the decoding performance of each model is given in Table 4.

**Table 4:** List of ROIs that have positive Shapley contributions to the decoding performance of each model.

Decoding model	ROI									
	L PMC	R PMC	L-DLPFC	R-DLPFC	L-FPA	R-FPA	L-IFG	R-SMG	R-SI	R-MI
Pre-DM (base)		+		+	+					+
Post-PM-30	+	+	+			+	+			
Post-PM-60	+	+	+	+	+	+				
Post-PM-90	+		+		+		+	+	+	
Post-MM-30	+		+	+	+		+	+		+
Post-MM-60		+				+			+	+
Post-MM-90						+		+		

## 4 Discussion

### 4.1 General Comments, Contribution, and Novelty of the Study

The aim of this study was to propose a TL-based DL methodology for accurate detection and objective classification of the neural processing of painful and non-painful stimuli that were presented at different timings post-analgesic or placebo drug administration. The motivation behind utilizing a TL approach relied on the assumption that such an adaptive training methodology would demonstrate a high performance while being computationally efficient and would remove the necessity to build new DL models for data collected at different clinical or daily life conditions for which obtaining training data is not practical and building a new model will have a computational cost. The feasibility of the TL approach was demonstrated by its efficacy in predicting the presence of painful or non-painful stimuli from non-invasive fNIRS recordings obtained under two different drug administrations and at three time points post-drug administration.

Each of the Post-DMs had mean accuracy, sensitivity, specificity, and AUC performances above 90% when the weights obtained from the base model were transferred, and no statistically significant differences in classification performance were found across the Post-DMs for any of the performance metrics. These results demonstrated that knowledge obtained from a pre-drug base model could be successfully utilized to build novel models for predicting the perceived pain intensity level from neurally induced hemodynamic signals obtained at six distinct dynamic brain states that were altered with either analgesic or a placebo intervention and at three different times post-drug administration.

The presented work includes several novelties. To date, there have been no studies that have demonstrated the efficacy of TL methodology for single-trial classification of the presence of painful and non-painful stimuli processing before and after morphine and placebo drug administration. This is the first study that tested the feasibility of integrating TL methodology with neurophysiological data obtained from a non-invasive, mobile, and wearable fNIRS system for the purpose of predicting pain and non-pain states under different drug administrations in healthy male adults. This approach was quite remarkable because it provides a proof of concept preliminary analysis that demonstrates the practicality of adapting a pre-drug base decoding model to different clinical conditions where collecting training data is not possible. This is the first study that explains the behavior of a pain decoding model both at pre-drug and post-drug conditions by utilizing an explainable artificial intelligence (AI) approach where the motivation was to understand which cortical regions contributed to the output of the model at most for every session.

The work introduces a convolutional neural network (CNN)-based TL approach and explainability analysis with DeepSHAP, which may be prominent for two potential applications: (1) a DL model trained with fNIRS data obtained during a base condition can be adapted to fNIRS data collected at different clinical or daily life conditions where obtaining training data may not feasible/practical to build novel ML or DL models. (2) Unveiling the explanation power of different cortical regions of interest may aid the design of more computationally efficient BCI system designs that target pain detection and such an approach may provide more precisely localized physiological markers of pain.

### 4.2 Comparison of the Classification Performances of DL-Based TL Methodology with ML techniques

We utilized a DL approach due to the fact that hand-crafted feature extraction via ML techniques may induce a bias when compared with DL techniques which generally do not require any domain knowledge. We had an initial test for exploring the general efficacy and feasibility of feeding various ML classifiers with well-known time domain features extracted from our dataset. Briefly, we performed classification of high and low pain conditions from ΔHbO and ΔHb responses separately by training several well-known machine learning algorithms with five conventional time domain BCI features extracted from each channel ΔHbO or ΔHb signal. In Supplementary Material 2, we reported the results of several different classifiers using different methods and parameters and our results revealed that ML algorithms showed less performance compared with our proposed DL model. DL algorithms perform feature selection by combining raw data into successively more complex and useful composite representations (please see Koppe et al.^41^ for a review). By creating these representations, the deep learner can increase its computational capacity to discover predictive functions with optimum efficiency. In this manner, it may maximize the predictive power provided by its input data, resulting in better performance compared with shallow architectures.^42^ Hand-crafted feature extraction for training ML algorithms may induce some bias or underperform due to the potential missing of some unincluded features which may have a prominent effect on the classification performance.

In the present study, we utilized only ΔHbO data for model development. We have three main rationales for not including information from ΔH data. First, we statistically compared SNR values of ΔHbO and ΔHb data and found that SNR values of ΔHbO are significantly higher than SNR values of ΔHb. Second, ΔHbO was reported as a more reliable indicator of cortical activation as the original authors of the dataset also mentioned in several studies conducted on the same dataset. These studies reported their ΔHbO results while excluding information from ΔHb data. Third, we tested our proposed DNN architecture with our ΔHb dataset and found that our network could reach a maximum training accuracy of 0.8 for the pre-drug model and 0.6 for validation which supported the notion that ΔHb data did not inherit the desired knowledge for discriminating pain and non-pain conditions. Meanwhile, the test accuracy was found to be 0.594±0.042. Such a low test accuracy implies that the pre-drug ΔHb model is not effective enough to transfer its knowledge to post-drug ΔHb datasets. The low classification performance obtained after applying the same methodology to ΔHb datasets could most probably be attributed to their low SNR. A statistical comparison of SNR values of ΔHbO and ΔHb data and accuracy curves of training and validation of the DNN model using ΔHb data are given in Supplementary Material 3.

In the presented work, we propose that utilizing whole time-series data information from multiple cortical regions during the processing of painful or non-painful stimuli will be more efficient when compared with extracting specific customized, hand-crafted features from the data as is performed with ML approaches. As given in Table 5, previous pain classification studies performed with ML approaches reported decoding accuracies between 67% and 94%. Among these studies, Lopez-Martinez and colleagues found the highest accuracy among these studies which was reported to be 94.17±4%. Compared with our study, Lopez-Martinez and colleagues focused on a more challenging problem which was a four-class classification task. However, they achieved this accuracy score using 25 features that were extracted from time, frequency, and wavelet domains. Feature extraction by performing these calculations requires an enormous manual effort. We should note that an objective comparison of our performance results with the performances reported in previous studies is complicated because the study designs differed in terms of sample size, number of classes, type and number of features, CV procedure, and the selected classifiers. Nonetheless, we can still conclude that the performance metrics achieved with our DL-based TL methodology fall in the high-performance spectrum among the performance metrics reported in previous pain classification studies.

**Table 5:** Comparison of the classification accuracies of fNIRS-based ML pain classification studies with the presented work.

Study	Task	Used features	Classifier	Performance
Lopez-Martinez et al.^4^	Electrical pain (high/low intensity)	Scalogram-based features (mean, maximum, and std. dev)	Hiearchical Bayesian logistic regression	Accuracy: 81%
Fernandez-Rojas et al.^5^	Temperature level (cold and hot)–based pain intensity (low and high)—four classes	25 features (time, frequency, and wavelet)	Gaussian SVM, KNN, and LDA	Gaussian SVM accuracy: 94.17%
Fernandez-Rojas et al.^6^	Temperature level (cold and hot)–based pain intensity (low and high)—four classes	Raw HbO and Hb	Bidirectional LSTM	Accuracy: 90.6%
Khan et al.^7^	Electrical pain (high/low intensity)	Log energy, crest factor, shape factor, impulse factor, margin factor, mobility, complexity, mean absolute deviation of the first difference, range, and variation in the first difference of HbO and Hb	SVM	Accuracy: 68.51%
Presented work	Electrical pain (high/low intensity)	Preprocessed epochs	CNN	Accuracy: 97%

How the challenges that exist for obtaining repeatable and reliable fNIRS signals across different post-drug sessions can be addressed with the implementation of DL techniques still remains an issue of debate. Major concerns associated with the implementation of DL techniques to fNIRS signals include habituation effects, variability in pain-induced hemodynamic response across subjects and sessions, and possible changes in the shape of pain-induced hemodynamic responses due to drug administration. Repeated exposure to the same stimuli may cause a reduction in hemodynamic response due to habituation effects which can significantly affect the reliability of fNIRS data.^32^ Such habituation effects may increase variability across subjects’ responses over time, leading to diminished neural and hemodynamic signals, which can impact the model’s generalizability and predictive power. Using a TL methodology may provide a solution for this problem because training the base model on pre-drug data is accomplished by the use of hemodynamic responses that are presumably more natural and less influenced by habituation. This allows the pre-drug base model to capture fundamental patterns associated with painful and non-painful stimuli. TL then adapts this model to post-drug conditions, maintaining its sensitivity to the original stimuli characteristics despite potential reductions in response due to habituation. This approach ensures that the model can generalize effectively from its initial training and remain robust against repeated exposures, thus mitigating the impact of habituation.^42^

DL models, especially those utilizing convolutional or recurrent neural network architectures, excel at handling high-dimensional data and capturing complex patterns that vary across individuals. These models can learn from the intrinsic variability present in the pre-drug fNIRS data, identifying consistent patterns across different subjects. The application of DeepSHAP further enhances this capability by providing insights into how different ROIs contribute to the classification decisions for both pre- and post-drug administration. This interpretative tool elucidates the contributions of specific ROIs to classification outcomes, highlighting variability in pain processing across individuals. Unlike mass univariate approaches DL models evaluate all variables in conjunction. This capability allows the models to capture the relationships and interactions among different features, leading to potentially more accurate and robust classifications of pain based on the comprehensive spatial patterns of neural activity, rather than isolated hotspots.^42^ The effects of analgesic drugs on the shape of hemodynamic response waveforms pose another significant challenge. TL is particularly crucial in this context. By initially training a model on pre-drug data and subsequently adapting it to data collected at various post-drug administration times, the DL model can adjust to changes in neural responses induced by the drug. This method involves learning the fundamental response patterns under normal conditions first then adapting to the shifts in these patterns due to drug effects. Moreover, DeepSHAP aids in quantifying changes in the importance of different ROIs due to drug effects. For instance, if a particular ROI becomes more or less responsive under analgesia, DeepSHAP can highlight this change, enhancing our understanding of how pain processing is altered by drugs.

### 4.3 Comparison of the Classification Performances of Pre-DM and Post-Drug Models

Test performances of Pre-DM and all Post-DMs achieved mean accuracy, specificity, sensitivity, and AUC scores above 90%. The mean performance of Pre-DM was above 95% for accuracy, sensitivity, specificity, and AUC metrics. Pre-DM had a statistically significantly higher two-class classification performance than all Post-MM models for all performance metrics, whereas Post-MM models did not demonstrate a significant difference among each other for any of the metrics. Similarly, Pre-DM had a statistically significantly higher two-class classification performance than all Post-PM models for all metrics, whereas Post-PM models did not demonstrate a significant difference among each other for any of the metrics.

Although the base model has a higher performance than post-drug models, the fact that all models have a general classification performance above 90% in all performance metrics demonstrates that knowledge obtained from a pre-drug base model could be successfully utilized to build novel models for predicting the pain intensity level from neurally induced hemodynamic signals. The performance of Post-MM models was relatively lower than that of Pre-DM and Post-PM models. This result is expected as morphine alters hemodynamic response patterns in several cortical regions including mPFC as shown in the previous work of Peng et al. (2018) from whom the dataset was obtained.

Neurophysiological and behavioral consequences of pharmacological interventions during acute^43^^–^^45^ and chronic pain^46^^–^^48^ conditions have been thoroughly examined in the last few decades. Recently, neural correlates of various drug interventions have been investigated using fMRI^49^^–^^51^ and fNIRS.^19^^,^^52^ Peng and colleagues^19^ conducted an fNIRS study that focused on the effect of placebo and morphine intervention on neural correlates of acute pain, and they reported that morphine attenuates hemodynamic response activity in the medial frontopolar area. In addition, Hansen and colleagues^49^ conducted an fMRI study that examined the neural effects of analgesic drugs (morphine/placebo) under acute painful stimulation, and although morphine-based attenuation was observed in the right insula, anterior cingulate cortex, and inferior parietal cortex, no difference in brain activation between pre- and post-placebo administration conditions was observed. These studies highlighted the fact that morphine administration alters cortical hemodynamic activity induced by neural processing of nociceptive stimuli. One potential reason for the relatively lower classification performance of Post-MM models may be due to the alterations of hemodynamic responses obtained during both pain and non-pain conditions in several brain regions with morphine administration with respect to the neural activity obtained during drug-free conditions. Wager and colleagues^51^ conducted a study that focused on extracting a pain signature. They combined fMRI measures with a machine learning method (least absolute shrinkage and selection operator regularized principal components regression—LASSO PCR) to classify the neural processing of painful and non-painful stimuli under remifentanil administration. Their proposed methodology achieved 90% sensitivity and 81% specificity before drug administration, and 86% sensitivity and 62% specificity were achieved after drug treatment. The study whose dataset was utilized in the presented work also found that morphine reduced the pain-induced hemodynamic responses in mPFC; however, it did not change the responses induced by non-painful stimuli. Meanwhile, the placebo drug affected the spatiotemporal patterns of neither painful nor non-painful induced hemodynamic responses.^19^

On the other hand, two-way ANOVA results demonstrated that the accuracy of Post-PM models was significantly greater than the decoding accuracy of Post-MM models. Chen^53^ provides an excellent review of the performance of different supervised and unsupervised classification algorithms in the correct identification of acute and chronic pain conditions by use of data obtained from different functional imaging modalities. In our work, the accuracy, sensitivity, and specificity performances of post-drug models were not statistically significantly different from each other per intervention type, whereas they remained in the high-performance spectrum among the performance metrics reported in previous studies that targeted two-class classification of pain intensity by use of functional neuroimaging measures.

### 4.4 Interpretation of Regional Shapley Contributions

#### 4.4.1 Pre-drug condition

In the pre-drug stage, R PMC, R DLPFC, L FPA, and R MI positively contributed to the highly accurate (97%) decoding performance of Pre-DM (Fig. 6 and Table 4). Among these regions, MI is a widely known and key region in pain processing which has a notable role in integrating sensory and motor aspects of pain.^54^^–^^56^ DLPFC is involved in several cognitive processes such as attention^57^^–^^59^ and working memory^60^ as well as neural processing of acute and chronic pain.^61^ Previous acute pain studies revealed that bilateral DLPFC activity has a negative correlation with the extent of the unpleasantness of thermal pain^62^ and pain catastrophizing scores.^63^ On the other hand, R DLPFC was found to be strongly associated with control of perceived pain intensity.^64^ The positive contribution of DLPFC to Pre-DM classification performance may be due to its involvement in the abovementioned cognitive aspects of pain experience.

R PMC (BA 6) was also found to be a positive contributor to the classification performance of Pre-DM. BA6 is a large cortical area located at the anterior side of MI and this region is primarily responsible for motor acts such as writing and speech besides sensory guidance of movement.^65^^,^^66^ A previous arterial spin labeling-MRI (ASL-MRI) study revealed that acute cold and heat pain resulted in an increase in cerebral blood flow (CBF)^67^ which could serve as a potential biomarker of acute pain. In a PET-based CBF study, PMC showed significant responses to both heat and cold pain in both genders.^68^ However, how PMC is effective in pain processing still remains unclear. Previous studies claimed that activity increase in PMC might be related to anticipation of movements to avoid painful stimuli.^69^ Contribution of R PMC to the classification performance of our Pre-DM model might be related to its role in regulating avoidance behavior for painful and non-painful stimuli.

L FPA (BA 10), a region located at the anterior portion of the PFC, was previously found to be a critical region in pain processing in previous fMRI^70^^–^^75^ and fNIRS^19^^,^^76^ studies. Previous reports suggest that FPA might be involved in collation, integration, and high-level processing of pain.^77^ In the study from which the dataset was generated, the statistically significant difference in hemodynamic responses to painful and non-painful stimuli was found in medial BA 10 of the pre-scan datasets of morphine and placebo visits of all subjects.^19^ The significant difference in hemodynamic responses to painful and non-painful stimuli might be the reason for the positive contribution of L FPA to the highly accurate Pre-DM decoding performance. The role of BA 10 in pain perception still remains unclear. However, anatomical connections exist between BA 10 and several cortical and subcortical regions such as the thalamus, insula,^78^^,^^79^ and anterior cingulate cortex (ACC)^80^^–^^82^ which play important roles in sensory discrimination and pain perception.^77^

#### 4.4.2 Post-drug condition

Among Post-PM models, L PMC, R PMC, L DLPFC, R FPA, and L IFG positively contributed to the classification performance of Post-PM-30 (Fig. 6 and Table 4). L PMC, R PMC, R DLPFC, L DLPFC, L FPA, and R FPA regions positively contributed to the classification performance of the Post-PM-60 model, and L PMC, L DLPFC, L FPA, L IFG, R SMG, and R SI positively contributed to the classification performance of the Post-PM-90 model. Regions that contributed both to Pre-DM and Post-PM models were R PMC and R DLPFC for Post-PM-30, R PMC and L FPA for Post-PM-60, and L FPA for Post-PM-90.

Common positively contributing regions to the classification performance of Pre-DM and Post-MM models were R PMC for Post-MM30; R PMC, R DLPFC, and L FPA for Post-MM60; and L FPA for Post-MM90. Despite these positive contributor regions common to both Pre-DM and Post-MM models, additional regions also contributed to the output of the Post-MM models. This observation may suggest that transferring knowledge from a pre-drug base model might be useful to decode the presence of a painful response; however, information from additional cortical regions may also be needed for high decoding performance in Post-DMs because of the intra- and intersubject variability introduced to fNIRS signals by efficacy duration of the analgesic drug.

A recent fMRI meta-analysis on placebo analgesia revealed that placebo administration causes small and widespread activity reductions during painful stimuli processing in several brain regions that are related to both painful stimulus and decision-making processes.^83^ DLPFC and PMC were found to be the common contributor regions across all developed models for Post-PM models. Among these regions, PMC and SMA were previously reported as critical regions which might reflect a placebo effect on pain-induced hemodynamic response.^67^ PMC activation was reported during painful stimulation under a high level of placebo administration.^84^ In the same study, a positive correlation was found between PMC and ACC activities, and ACC activity is strongly related to placebo and opioid analgesia.^85^^,^^86^ Changes in the hemodynamic activity in PMC might be associated with the hemodynamic activity in ACC which cannot be measured using fNIRS. On the other hand, DLPFC plays a role in pain suppression by attention-based pain regulation,^62^^,^^87^^–^^89^ and it was particularly involved in placebo analgesia.^90^^,^^91^ Previous studies also reported that there was a correlation between DLPFC connectivity and placebo analgesia.^92^^,^^93^ We think that DLPFC positively contributed to pain decoding during placebo analgesia due to its pain regulatory role.

Similarly, FPA (BA 10) was also found to be another region that positively contributed to the decoding performance of Post-PM models. Previous evidence suggests that FPA plays a role in pain anticipation under placebo analgesia^94^^–^^96^ and increased activation in FPA during pain expectation might be related to placebo analgesia and emotional regulation.^94^ Compared with Amanzio et al., L IFG presented a placebo-induced activation increase in another meta-analysis^97^ and is considered a critical anticipatory predictor of placebo analgesia.^98^ We think that both these regions contributed to decoding performance due to their regulation and anticipation roles during the placebo analgesia condition. Previous evidence related to the behavior of SI showed that pain-induced activity decreased after placebo analgesia.^85^^,^^99^^,^^100^ However, in the previous study of this dataset, no significant difference was reported in SI between painful and non-painful stimuli when compared with pre-drug status.^19^ In that study, due to not having any comparison between painful and non-painful stimuli for each drug condition, it is hard to make a direct interpretation related to the reason for the contribution of SI. Statistical similarity does not fully guarantee a low-accurate discrimination of two classes using ML approaches.^101^ On the other hand, SMG is located at the inferior parietal lobule which is involved in pain relief.^83^^,^^102^ Wager and colleagues^51^ found that SMG is a positive predictor of decoding painful versus non-painful stimulus.

For Post-MM, regions that positively contributed to the decoding performance were L PMC, L DLPFC, R DLPFC, L FPA, L IFG, R SMG, and R MI after 30 min of administration. R PMC, R FPA, R SI, and R MI positively contributed to the model after 60 min of administration, and R FPA and R SMG positively contributed to the model after 90 min of administration. Effects of opioids such as morphine and its derivatives such as remifentanil on painful stimuli have previously been investigated in several fMRI^49^^,^^51^^,^^103^^–^^105^ and fNIRS^19^ studies. Morphine-induced activation reduction was observed in DLPFC,^103^ inferior parietal lobe which covers SMG,^49^^,^^103^ and FPA.^19^ Among these regions, a previous MR spectroscopy study revealed that the frontal region is an opioid-rich region^106^ which is possibly effective in reducing perceived pain intensity. On the other hand, although pain-induced hemodynamic activity reduction in SI was observed after morphine administration,^19^^,^^104^ no difference was found between pre-morphine and post-morphine non-painful stimuli induced hemodynamic activity in SI.^19^^,^^103^

### 4.5 Interpretation of Violin Plots

The comparative illustration of the distribution of classification performance metrics of Pre-drug and Post-drug models in Fig. 5 highlights several considerations. A key observation is the broad range of values in the plots corresponding to the 60-min post-morphine administration which might be indicative of the peak effect time of the drug. The analgesic effect of morphine is considered to be most pronounced around this time point, which in turn might lead to significant physiological variability among subjects.^19^ More specifically, the wide distribution during the peak effect period of morphine may reflect the diverse physiological responses to the drug among subjects. Such variability may impact the predictive capabilities of the transferred model knowledge, causing fluctuations in metrics such as accuracy, sensitivity, and specificity. Given that the model was initially trained on pre-drug data, it appears less equipped to handle the sudden physiological changes accompanied by drug administration. This situation highlights the need for incorporating a broader range of data in the training phase which can capture various physiological states during drug effects. On the other hand, morphine administration has been shown to attenuate pain-induced HbO signals at some cortical regions involving the anterior portion of the frontal cortex^19^ and make the distinction between painful and non-painful stimuli-induced HbO signals harder.

Morphine may also reduce sensory and affective neural responses to painful stimuli. This effect may in turn make differences between neural processing of painful and non-painful stimuli smaller and make their classification harder. Indeed, morphine may inhibit nociceptive processing at cortical and subcortical regions that are responsible for sensory and emotional regulation [e.g., the insula, the somatosensory cortex (the primary somatosensory cortex, S1, and the secondary somatosensory cortex, S2), and the ACC].

Placebo drug administrations are known to have modulatory emotional, anticipatory, and motivational effects. Similar to morphine administration, placebo administration may reduce sensory and affective neural responses to painful stimuli as observed in the study of Peng et al. (2018) which may induce suppression or attenuation of pain-induced HbO signals in the mPFC. More specifically, placebo drug administration may inhibit nociceptive processing at the cortical and subcortical regions that are responsible for sensory and emotional regulation [e.g., the insula, the somatosensory cortex (the primary somatosensory cortex, S1, and the secondary somatosensory cortex, S2), and ACC]. Hence, post-placebo drug conditions may not be identical to the pre-drug state in terms of sensory and emotional assessment of painful stimuli.

The differences in identified brain regions between the pre-drug model and various post-placebo models can be attributed to the placebo effect which involves individual variability in psychological and physiological responses and the neural mechanisms underlying pain perception and modulation. The placebo effect, influenced by the therapeutic context, can lead to genuine psychobiological changes affecting brain activity (Finniss et al.,^107^ 2010, Klinger et al.,^108^ 2014). In a 2004 fMRI study by Wager et al., changes in pain perception induced by the use of a placebo were demonstrated. In placebo-administered subjects, hemodynamic signals responding to the pain signal underwent changes due to the anticipation effect generated by the frontal cortical regions. In our study, the difference in classification performance observed after applying the weights of models developed with pre-drug data to post-placebo data using a TL approach can be explained by the fact that the signals obtained during pre-drug and post-placebo sessions are quite similar but not exactly equal. This situation demonstrates some resemblance to the domain-shift problem that arises in deep learning studies. Although the transfer learning method is adapted to eliminate this problem, a certain amount of performance loss is expected due to the insufficient amount of training data. In addition, the expectation of pain relief can produce alterations in pain processing regions over time.^109^ These factors, coupled with the complex interplay of ascending nociceptive and descending inhibitory pathways, result in different brain regions being involved in pain decoding at different post-placebo intervals.^110^ This variability may underscore the dynamic nature of pain perception and the significant role of psychological factors in modulating pain responses.

In the context of deep learning and transfer learning, the pre-drug model was trained on relatively stable physiological data. Introducing placebo data introduces unexpected variability, which the model must adapt to, potentially resulting in differences in performance metrics. This challenge highlights the necessity of incorporating diverse training data that captures a range of physiological states to enhance model robustness.

### 4.6 Potential of the Proposed Methodologies

The presented work demonstrated that knowledge obtained from a pre-drug base model could be successfully transferred to build novel models for predicting pain and non-pain states from neurally induced hemodynamic signals obtained at six distinct dynamic brain states which were altered with either morphine or a placebo drug administration at three different timings post-drug administration. We provide a proof of concept preliminary analysis that demonstrates the practicality of adapting a pre-drug base decoding model to different clinical conditions where collecting training data is not possible. The low computational cost and high classification performance of the TL approach make it feasible for specific classification problems where baseline data are available and a model trained with this baseline data can be adapted to data collected at different clinical or daily life conditions where obtaining training data is not feasible/practical to build novel ML or DL models.

Unveiling the explanation power of features obtained from different cortical regions of interest is prominent as it may aid the design of more computationally efficient BCI system designs that target pain detection and such an approach may provide more precisely localized physiological markers of pain. In the presented work, Shapley values presented no consistent localization of positive contribution across all models. Nonetheless, the proposed combination of TL-based DL methodology with an xAI method and their application to fNIRS data demonstrate a potential for unveiling the explanation power of different ROIs and this analytical procedure may aid the design of more computationally efficient BCI system designs for other application areas.

### 4.7 Limitations of the Study and Recommendations for Future Work

Pain is a multisensory experience, and test–retest reliability is always questionable in human functional neuroimaging studies that target cognitive and emotional aspects. We should not ignore the fact that pain-responsive cortical areas do not solely process pain-induced neural information. Both morphine and placebo interventions result in different cognitive and anticipation effects and decoding the intensity of a painful stimulus and its saliency dimension cannot be differentially performed.^111^ Due to these constraints, we should take into account the fact that the relative contribution of morphine- and placebo-modulated regions may show variability within and across participants and across different pharmacological conditions. Painful and non-painful stimuli may have different hemodynamic activation strengths at each post-drug session which may be not only due to the differential effect of the type of drug administration but also due to the varying cognitive state at each session including habituation effects.

Although the number of subjects in our study was comparable to the sample sizes reported in previous pain decoding studies, the low number of subjects was another critical limitation of our study. DL algorithms require high amounts of data for training.^112^ However, obtaining comparably high numbers of labeled data in the medicine field is difficult due to factors such as acquisition cost and labor. Hence, clinical studies are conducted with the relatively limited amount of data when compared with other areas of DL applications. To overcome this limitation, we applied a data augmentation procedure during model training by utilizing well-accepted data augmentation approaches.^36^ Nonetheless, although synthetic data are expected to capture the diversity and variability available in real-world data, its creation is still a biased approach. For best-case scenarios, training DL algorithms with more real-world data from more participants will increase the reliability of validation and reproducibility of our results. Besides, although DL methodologies remove the necessity of feature engineering and domain knowledge requirements, it should not be neglected that they still have many unknown parameters and require vast amounts of labeled samples for training.

Another critical limitation of the study is the information leakage that occurred as a result of performing a GLM-based short channel regression over a whole run of every single participant. After performing the short channel regression, we extracted high and low pain epochs for every participant, and we pooled them at the group level and separated them into training, test, and validation datasets. We had to prefer this approach over a leave-one-subject-out cross-validation procedure due to the low number of trials that would be available for each participant (six low pain and six high pain). Training our model using the trials of 13 participants and testing the model using 12 trials of a single participant would yield biased accuracy results due to the low number of available test data. By performing 30 runs of hold-out cross-validation (60% training, 20% test, and 20% validation sets), we were able to validate and test our trained models with a higher number of samples. A previous study recommended that reasonable precision for the validation of a classifier can be realized with a test sample of 75 to 100.^113^ Similarly, previous studies also used k-fold cross-validation rather than leave-one-out-subject cross-validation for pain decoding.^20^^,^^21^

## 5 Conclusion

The presented work addressed two main research questions. Our first question aimed to assess the feasibility of implementing a TL methodology to decode the neural processing of painful and non-painful stimuli obtained under two distinct pharmacological interventions and at different post-intervention times. Our results demonstrated that the neural processing of painful and non-painful stimuli states could be successfully distinguished by utilizing hemodynamic information obtained before and after a morphine or a placebo drug administration. The performance of the TL approach in the accurate classification of pain and non-pain states was tested on six distinct post-models which were fine-tuned for fNIRS data recorded during noxious and innocuous stimuli under different pharmacological conditions. Our results demonstrated the potential of training models with a baseline fNIRS data and adapting these baseline models to data collected at different clinical or daily life conditions where obtaining training data is not feasible/practical to build novel ML or DL models. Our second aim was to assess the contribution of features obtained from different cortical regions to the classification performance of the proposed DL model and how this contribution changes as hemodynamic activity is modified with morphine or placebo intervention. Our findings demonstrate the potential of the proposed methodology for unveiling the explanation power of different ROIs and how this approach may aid the design of more computationally efficient fNIRS-based BCI system designs for other daily life and clinical application areas.

## Supplementary Material







## Data Availability

In addition to HomER3, Tensorflow toolkit (version 2.8.0), and Shap toolbox, the code can be downloaded from the website https://github.com/aykuteken/Pain_decoding/. The data information can be found at https://www.nitrc.org/projects/yucel18pain/.
