# Supplementary material for: Explainable fNIRS-based pain decoding under pharmacological conditions via deep transfer learning approach
Source: Neurophotonics. 2024 Dec 17;11(4):045015. doi: 10.1117/1.NPh.11.4.045015 (PMC11651663; doi:10.1117/1.NPh.11.4.045015)
Supplement: Supplementary file 1 [file NPh_011_045015_SD001.pdf]

	Pre Drug Session					Morphine Session					Placebo Session				
**Channel Number**	Mean X	Mean Y	Mean Z	Std. Dev.	**Corr. Reg.**	Mean X	Mean Y	Mean Z	Std. Dev.	**Corr. Reg.**	Mean X	Mean Y	Mean Z	Std. Dev.	**Corr. Reg.**
**1**	-63.45	4.05	25.05	5.25	**L PMC**	-63.58	3.69	24.58	5.34	**L PMC**	-63.31	4.41	25.51	5.16	**L PMC**
**2**	-53.77	10.19	43.02	5.50	**L DLPFC**	-54.11	10.53	42.11	5.65	**L DLPFC**	-53.44	9.85	43.92	5.35	**L PMC**
**3**	-59.52	17.76	19.86	5.15	**L IFG**	-59.75	17.17	19.11	5.17	**L IFG**	-59.28	18.36	20.62	5.13	**L IFG**
**4**	-56.50	29.08	14.62	5.78	**L IFG**	-57.00	28.39	13.75	5.73	**L IFG**	-56.00	29.77	15.49	5.83	**L IFG**
**5**	-50.90	23.54	38.03	5.40	**L DLPFC**	-51.31	23.75	37.00	5.38	**L DLPFC**	-50.49	23.33	39.05	5.41	**L DLPFC**
**6**	-47.47	33.66	34.12	5.62	**L DLPFC**	-48.19	33.61	32.83	5.75	**L DLPFC**	-46.74	33.72	35.41	5.48	**L DLPFC**
**7**	-52.44	40.15	9.17	5.91	**L DLPFC**	-53.03	39.22	8.19	5.77	**L DLPFC**	-51.85	41.08	10.15	6.04	**L DLPFC**
**8**	-42.99	44.76	28.61	5.79	**L DLPFC**	-43.83	44.53	27.39	5.85	**L DLPFC**	-42.15	45.00	29.82	5.72	**L DLPFC**
**9**	-19.56	69.51	14.11	5.81	**L FPA**	-19.94	69.67	13.17	5.44	**L FPA**	-19.18	69.36	15.05	6.17	**L FPA**
**10**	-17.47	58.52	35.89	5.43	**L DLPFC**	-17.81	58.92	35.11	5.42	**L DLPFC**	-17.13	58.13	36.67	5.43	**L DLPFC**
**11**	-6.25	70.57	13.36	6.24	**L FPA**	-6.75	71.19	12.61	5.89	**L FPA**	-5.74	69.95	14.10	6.59	**L FPA**
**12**	9.98	70.96	14.35	6.61	**R FPA**	8.58	71.19	13.53	6.45	**R FPA**	11.39	70.72	15.18	6.77	**R FPA**
**13**	-5.52	61.40	35.19	5.78	**L DLPFC**	-5.83	62.00	34.58	5.64	**L FPA**	-5.21	60.80	35.80	5.91	**L DLPFC**
**14**	7.95	60.90	35.92	6.33	**R DLPFC**	6.36	61.50	35.17	6.21	**R DLPFC**	9.54	60.31	36.67	6.45	**R DLPFC**
**15**	24.39	68.41	15.18	6.45	**R FPA**	22.78	69.25	14.97	5.92	**R FPA**	26.00	67.56	15.39	6.98	**R FPA**
**16**	21.01	57.21	37.25	6.02	**R DLPFC**	19.50	58.17	37.06	5.32	**R DLPFC**	22.51	56.26	37.44	6.71	**R DLPFC**

**17**	36.39	-8.49	68.76	5.85	**R PMC**	34.86	-6.61	69.17	5.25	**R PMC**	37.92	-10.36	68.36	6.45	**R PMC**
**18**	38.04	-29.90	71.17	5.26	**R MI**	36.78	-27.75	72.11	4.24	**R MI**	39.31	-32.05	70.23	6.28	**R SI**
**19**	45.37	-8.26	63.41	4.88	**R PMC**	44.72	-6.53	63.22	4.84	**R PMC**	46.03	-10.00	63.59	4.92	**R PMC**
**20**	54.51	-8.50	55.52	5.03	**R PMC**	53.81	-6.56	55.78	4.61	**R PMC**	55.21	-10.44	55.26	5.44	**R PMC**
**21**	46.96	-29.95	65.71	4.30	**R SI**	46.69	-28.14	66.19	4.07	**R SI**	47.23	-31.77	65.23	4.53	**R SI**
**22**	56.01	-31.63	57.72	4.64	**R SMG**	55.56	-29.64	58.28	4.29	**R SMG**	56.46	-33.62	57.15	4.99	**R SMG**
**23**	61.34	-9.85	46.81	5.49	**R PMC**	61.11	-8.17	46.64	4.70	**R PMC**	61.56	-11.54	46.98	6.27	**R PMC**
**24**	63.14	-33.60	49.70	5.20	**R SMG**	63.61	-31.94	49.61	4.37	**R SMG**	62.67	-35.26	49.80	6.02	**R SMG**

Table S1. Mean MNI coordinates of long channels averaged across all subjects and scans for pre-drug, morphine and placebo administration sessions. The corresponding anatomical regions are given in Talairach Space. Std. Dev. : Standard Deviation, Corr. Reg. : Corresponding Region, L: Left, R: Right, PMC: Pre-motor cortex, IFG: Inferior Frontal Gyrus, SI : Primary Somatosensory Cortex, MI: Primary Motor Cortex, SMG: Supramarginal Gyrus, FPA: Frontopolar area, DLPFC: Dorsolateral prefrontal cortex.
