# Supplementary material for: Explainable fNIRS-based pain decoding under pharmacological conditions via deep transfer learning approach
Source: Neurophotonics. 2024 Dec 17;11(4):045015. doi: 10.1117/1.NPh.11.4.045015 (PMC11651663; doi:10.1117/1.NPh.11.4.045015)
Supplement: Supplementary file 2 [file NPh_011_045015_SD002.pdf]

SupplementaryFile-2

The selected time domain features were mean, peak value, variance, skewness and kurtosis. Hence, a total of 120 features from 24 channels were utilized to model hemodynamic responses corresponding to each stimulus during each drug condition. After performing z-score normalization to the feature sets, we first performed PCA to reduce the dimension by considering the 95% of explained variance. Then, we followed a 10-fold cross-validation procedure to cope with the overfitting problem. We compared the classification performances of decision tree, SVM (Linear, Polynomial, RBF kernels), KNN, ensemble classifier and ANN algorithms with this quasi-standard pipeline. We utilized Classification Learner toolbox of MATLAB to solve our 2 class classification problem. In the table below, initial classification results revealed that the machine learning based methods did not demonstrate promising performances that could be further improved to accuracy scores above 90 % with further tuning while the proposed transfer learning based deep learning method resulted in accuracy scores above 90% for all drug conditions.

Table S2. Machine learning results for ΔHbO and ΔHb datasets obtained for each drug condition. Bold and underlined values are the highest accuracy values across all classifiers (SVM: Support Vector Machine, KNN: K-nearest neighborhood, LDA: Linear discriminant analysis, QDA: Quadratic discriminant analysis, DT: Decision trees, LR: Logistic Regression).

	Pre Drug Condition		Post Morphine						Post Placebo					
			30 Min.		60 Min.		90 Min.		30 Min.		60 Min.		90 Min.	
Classifier	Pre-Drug ΔHbO	Pre-Drug ΔHb	PM-30 ΔHbO	PM-30 ΔHb	PM-60 ΔHbO	PM-60 ΔHb	PM-90 ΔHbO	PM-90 ΔHb	PP-30 ΔHbO	PP-30 ΔHb	PP-60 ΔHbO	PP-60 ΔHb	PP-90 ΔHbO	PP-90 ΔHb
DT (Fine)	51.5	39.4	65.7	45.7	41.9	48.4	60.0	**71.4**	54.8	38.7	40.0	45.7	60.0	50.0
DT (Medium)	51.5	42.4	65.7	45.7	41.9	48.4	60.0	**71.4**	54.8	38.7	40.0	45.7	60.0	50.0
DT (Coarse)	63.6	47.0	60.0	51.4	35.5	58.1	51.4	48.6	41.9	45.2	45.7	**60.0**	63.3	53.3
LDA	68.2	47.0	77.1	57.1	58.1	58.1	48.6	54.3	38.7	51.6	42.9	51.4	63.3	53.3
QDA	54.5	37.9	74.3	45.7	48.4	41.9	54.3	68.6	51.6	35.5	**57.1**	54.3	63.3	53.3
LR	63.6	45.5	74.3	51.4	54.8	61.3	48.6	51.4	35.5	45.2	37.1	57.1	46.7	43.3
NB (Gaussian)	65.2	48.5	71.4	60.0	54.8	61.3	42.9	42.9	51.6	38.7	48.6	54.3	70.0	46.7
NB (Kernel)	65.2	39.4	62.9	62.9	67.7	51.6	60.0	54.3	45.2	45.2	**57.1**	45.7	66.7	46.7
SVM (Linear)	63.6	43.9	**82.9**	60.0	51.6	54.8	51.4	51.4	35.5	51.6	42.9	48.6	73.3	50.0
SVM (Quadratic)	62.1	37.9	80.0	57.1	61.3	54.8	57.1	54.3	38.7	38.7	40.0	54.3	76.7	53.3
SVM (Cubic)	60.6	39.4	77.1	54.3	67.7	58.1	54.3	51.4	54.8	**64.5**	40.0	54.3	66.7	56.7
SVM (Fine Gaussian)	53.0	39.4	65.7	48.6	54.8	54.8	54.3	48.6	48.4	54.8	40.0	54.3	66.7	60.0
SVM (Medium Gaussian)	62.1	45.5	77.1	60.0	54.8	58.1	54.3	54.3	38.7	45.2	40.0	54.3	76.7	50.0
SVM (Coarse Gaussian)	50.0	50.0	48.6	48.6	54.8	48.4	51.4	48.6	38.7	48.4	48.6	48.6	66.7	53.3
KNN (Fine KNN)	48.5	37.9	60.0	57.1	58.1	51.6	42.9	42.9	**61.3**	45.2	34.3	54.3	73.3	50.0
KNN (Medium KNN)	40.9	47.0	60.0	54.3	48.4	58.1	48.6	68.6	48.4	54.8	40.0	68.6	76.7	53.3
KNN (Coarse KNN)	54.5	**53.0**	51.4	48.6	54.8	54.8	57.1	51.4	51.6	48.4	**57.1**	45.7	56.7	50.0

KNN (Cosine KNN)	60.6	47.0	74.3	48.6	51.6	54.8	57.1	60.0	51.6	51.6	40.0	51.4	76.7	60.0
KNN (Cubic KNN)	53.0	48.5	54.3	48.6	51.6	54.8	45.7	65.7	48.4	51.6	40.0	**60.0**	56.7	56.7
KNN (Weighted KNN)	47.0	34.8	68.6	62.9	48.4	54.8	57.1	68.6	54.8	54.8	37.1	**60.0**	66.7	63.3
Ensemble (Boosted Trees)	62.1	42.4	48.6	51.4	51.6	48.4	45.7	48.6	51.6	51.6	42.9	45.7	50.0	50.0
Ensemble (Bagged Trees)	59.1	40.9	65.7	57.1	54.8	51.6	57.1	54.3	35.5	54.8	48.6	48.6	73.3	46.7
Ensemble (Subspace Discriminant)	**69.7**	45.5	77.1	57.1	58.1	58.1	48.6	54.3	38.7	51.6	42.9	51.4	60.0	53.3
Ensemble (Subspace KNN)	56.1	31.8	74.3	**62.9**	58.1	51.6	57.1	62.9	38.7	41.9	37.1	51.4	**76.7**	50.0
Ensemble (RUSBossted Trees)	51.5	40.9	62.9	45.7	51.6	45.2	54.3	68.6	51.6	51.6	37.1	48.6	50.0	50.0
Neural Network (Narrow)	56.1	31.8	68.6	54.3	61.3	58.1	54.3	54.3	51.6	45.2	42.9	45.7	63.3	56.7
Neural Network (Medium)	59.1	33.3	77.1	51.4	54.8	**64.5**	54.3	54.3	41.9	51.6	48.6	54.3	63.3	43.3
Neural Network (Wide)	59.1	34.8	71.4	62.9	67.7	54.8	54.3	51.4	51.6	54.8	42.9	51.4	56.7	46.7
Neural Network (Bilayered)	53.0	33.3	74.3	60.0	61.3	58.1	51.4	51.4	45.2	61.3	42.9	57.1	73.3	43.3
Neural Network (Trilayered)	60.6	30.3	65.7	54.3	54.8	54.8	**65.7**	57.1	54.8	54.8	42.9	**60.0**	70.0	33.3
Kernel (SVM Kernel)	59.1	47.0	77.1	51.4	**71.0**	54.8	57.1	48.6	45.2	48.4	45.7	54.3	66.7	**70.0**
Kernel (Logistic Regression Kernel)	57.6	39.4	80.0	45.7	67.7	61.3	62.9	51.4	45.2	48.4	45.7	45.7	**76.7**	60.0
