# Supplementary material for: Explainable fNIRS-based pain decoding under pharmacological conditions via deep transfer learning approach
Source: Neurophotonics. 2024 Dec 17;11(4):045015. doi: 10.1117/1.NPh.11.4.045015 (PMC11651663; doi:10.1117/1.NPh.11.4.045015)
Supplement: Supplementary file 3 [file NPh_011_045015_SD003.pdf]

We extracted SNR values corresponding to chromophore specific epoch hemodynamic data of each stimulus period for each channel, stimulus type and subject with the following formula:

$$SNR = \frac{\sigma_{task}^2}{\sigma_{rest}^2}$$

Where σ_{task}^2 and σ_{rest}^2 represents variance of the chromophore specific hemodynamic signal during task rest respectively. We performed a Shapiro-wilk test to check the normality of both ΔHbO and ΔHb datasets and found that they violated the normality assumption. Hence, we statistically compared the SNR of both chromophores with a Wilcoxon Sign Rank Test.

In Table S3, we demonstrated the statistical differences between SNR values of each chromophore for each channel for a representative session. For each channel, we extracted 336 SNR values obtained from 12 stimulus trials of 14 subjects after 2 sessions for ΔHbO and ΔHb time traces. We highlight the channels where SNR value of ΔHbO is statistically significantly higher than ΔHb for Post 90 Min. Drug Condition involving both placebo and morphine sessions.

Table S3. SNR comparison results of ΔHbO and ΔHb. Bold highlighted channels shows that SNR of ΔHbO is greater than SNR of ΔHb

	Post 90 Min. Data
Ch1	Z = -0.8934,p = 0.3717
Ch2	Z = -0.5705,p = 0.5683
Ch3	Z = -0.0412,p = 0.9671
Ch4	Z = 0.5526,p = 0.5805
Ch5	Z = 0.6725,p = 0.5012
Ch6	Z = 0.9481,p = 0.3431
Ch7	Z = 1.3067,p = 0.1913
Ch8	Z = 1.4982,p = 0.1341
Ch9	Z = 1.8147,p = 0.0696
**Ch10**	**Z = 2.9245,p = 0.0034**
**Ch11**	**Z = 3.4521,p = 0.0006**
**Ch12**	**Z = 3.4008,p = 0.0007**
**Ch13**	**Z = 2.8063,p = 0.005**
**Ch14**	**Z = 3.0485,p = 0.0023**
**Ch15**	**Z = 3.8389,p = 0.0001**
**Ch16**	**Z = 4.5521,p = 0**
**Ch17**	**Z = 3.8799,p = 0.0001**
**Ch18**	**Z = 3.0883,p = 0.002**
**Ch19**	**Z = 2.2926,p = 0.0219**
**Ch20**	**Z = 2.0482,p = 0.0405**

Ch21	Z = 0.5076,p = 0.6117
Ch22	Z = 0.2067,p = 0.8363
Ch23	Z = 0.1577,p = 0.8747
Ch24	Z = 0.6281,p = 0.5299

On the other hand, we performed training and validation over our proposed network by using the ΔHb data and we found that for pre-drug model, our network reaches maximum training accuracy as 0.8 and for validation 0.60. On the other hand, test accuracy was found as 0.594 ± 0.042. Such a low accuracy means that pre-model is not enough effective to transfer its knowledge for HbR data. These results showed that our approach does not seem valid for ΔHb due to low SNR. In Figure S3, training and validation curves of pre-drug model by using ΔHb are shown.

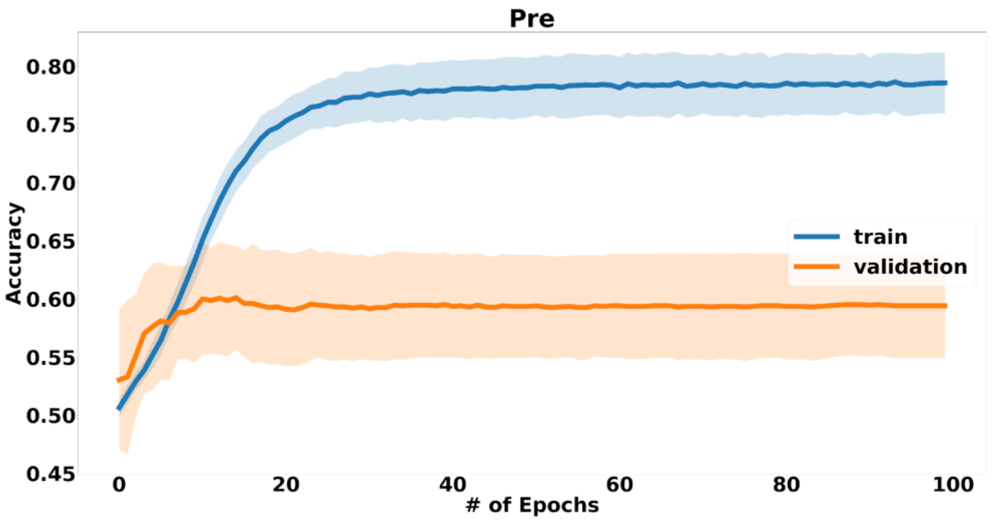

Figure S3. Training and Validation accuracy curves of proposed DNN architecture using ΔHb dataset
